# Editorial: The Implications of Transport Practices for Horse Health and Welfare

**DOI:** 10.3389/fvets.2020.00202

**Published:** 2020-04-21

**Authors:** Barbara Padalino, Christopher B. Riley

**Affiliations:** ^1^Division of Animal Sciences, Department of Agricultural and Food Sciences, Alma Master Studiorum, University of Bologna, Bologna, Italy; ^2^School of Veterinary Science, Massey University, Palmerston North, New Zealand

**Keywords:** horse, transportation, welfare, health, education

Animals are transported frequently for many different reasons, from competing to slaughter, over short or long distances during their lifetimes. However, transportation is a significant stressor for animals and frequently leads to severe behavioral and health problems (1–3). Transportation has therefore to be considered as a human-related risk for our animals (4, 5). While movements of several livestock species are traceable, in most countries horses can be moved without official communications or documentation. The outcome of this lack of process and procedure is that there are marked gaps in our knowledge of their implications. If one only considers the high performance horses competing for Fédération Equestre Internationale (about 80,000 horses), there are ~4,000 international events encompassing eight disciplines in 134 countries, each requiring extensive and frequent movements. In contrast, if one was to consider horses traveling for slaughter, most complete a single journey (6), but conservatively 800,000 horses are slaughtered annually globally. Consequently, transportation of performance, pleasure and slaughter horses is very common. In recognition of animal transportation as one or the persistent animal welfare concerns of our era, this special issue aims to educate all people involved with equine transport (owners, stakeholders, caretakers, drivers, veterinarians, and scientists) on the adverse consequences of transportation, associated risk factors, regulations and best practices. The articles encompassed within this Research Topic provide evidence in support of the need to safeguard horse welfare during transportation, and since transport also poses a biosecurity risk, there are suggestions on how to minimize disease spread.

In the study reported by Roy et al., the incidence of non-visible transport-related injuries in meat horses transported in Canada was of 54%, counting bruising of carcasses post-mortem. Unfortunately digital infrared thermography showed modest sensitivity to bruise detection after transportation. The authors suggest the need of find alternative ways to monitor the incidence of injuries and poor welfare due to long distance transportation prior to the slaughter of horses. A possible way to minimize injuries and behavioral problems during loading of horses destined for meat was suggested by Dai et al. Positive reinforcement training for self-loading resulted in a shorter loading times, a lower frequency of stress-related behavior and no injuries in young horses destined to slaughter. This training requires a short time to perform and modest commitment by horse handlers, but reduces loading time, contact between human and horses, and possible risk for both during loading. Considering that loading behavioral problems are risk factors for injuries (7), the targeting training described by Dai et al. should be strongly recommended to reduce evident and non-evident injuries occurring during the last journey toward the slaughterhouse. Improper training practice and lower level of experience in horse handling was confirmed as a risk factor for transport-related injuries also in the study of Padalino et al. In the latter study, the incidence of transport-related injuries in sport horses was lower (about 17%) than that reported by Roy et al. in slaughter horses. However, there were similarities among the types of injuries; the majority of the problems were indeed bruises as reported in Canada. However, in New Zealand performance horses reported also severe injuries (4%) requiring euthanasia. In this online survey conducted by Padalino et al., more than 1,100 responses were analyzed, and surprisingly the majority of the injuries happened in transit, rather than at loading or unloading. In 50% of the cases, horses and their behaviors were implicated as the possible cause. However, the study by Padalino et al. shows that human-related factors, in particular the background and education of horse handlers, are equally important as risk factors for transport-related injuries. In particular, since professionals are more exposed to the risk in particular those with <5 years of experience in horse handling, education on horse handling and focused animal transport training are recommended.

Injuries are probably the most frequent reported consequence of horse transport, however respiratory diseases are often considered the most serious complications (4, 8). Even if transport pneumonia is one of the most studied transport-related pathologies (9, 10), it is still difficult to identify horses at risk of developing this pathology. The retrospective study of Maeda and Oikawa documented the patterns of rectal temperature of horses transported over different distances (1,492–2,921 km) showing that horses tended to become pyretic between the 20th and 49th hours of transport, but some of them were not pyretic on arrival. Horses suffering from fever in transit had pathological alterations of their lungs. Maeda and Oikawa therefore, provide supporting evidence of the importance of monitoring the rectal temperature of horses during the journey to identify horses with subclinical pneumonia. Finally, the study of Muscat et al. investigates the possible role of equid herpesvirus in the development of transport-pneumonia. Surprisingly, in the latter study, clinical evidence of EHV-1 and EHV-4 was not detected, yet transportation led to increased shedding, transmission and reactivation of EHV-2 and EHV-5. The clinical significance of these viruses remain in question, but their role as suppressive immunomodulators should be taken into account.

The articles published in this Research Topic have added to our knowledge of equine transportation and have provide evidence that supports efforts to improve the current guidelines on animal welfare during transport. People involved in equine transport should be trained on best practices, in particular new stress free handling and evidence-based training underpinned by animal learning theory should be implemented, horse temperature, and behavior should be monitored continuously during and after the journey. However, as all studies do, these works raise more questions that need to be addressed in future studies. We are proud and highly motivated to promote the development of evidence-based guidelines in animal transportation in the Frontiers open source venue, and future article collections are needed to further address the topic of animal health and welfare during transportation.

## Author Contributions

All authors listed have made a substantial, direct and intellectual contribution to the work, and approved it for publication.

## Conflict of Interest

The authors declare that the research was conducted in the absence of any commercial or financial relationships that could be construed as a potential conflict of interest.
